# Safe retrieval of gastrointestinal foreign bodies using the novel endoscopic attachment with a flower-shaped design

**DOI:** 10.1055/a-2817-2822

**Published:** 2026-03-09

**Authors:** Sara Haruki, Takehiko Koga, Norihiro Kojima, Hiroshi Takamori

**Affiliations:** 1 91376 Internal Medicine/General Practice, Taragi Municipal Hospital, Kumamoto, Japan; 2 38208 Department of Gastroenterology and Medicine, Fukuoka University Faculty of Medicine, Fukuoka, Japan; 3 91376 Department of Gastroenterology, Taragi Municipal Hospital, Kumamoto, Japan; 4 91376 Surgery, Taragi Municipal Hospital, Kumamoto, Japan

When performing the endoscopic retrieval of foreign bodies from the gastrointestinal tract, attaching a hood to the distal tip of the endoscope is necessary to ensure safe removal 1 2 3 . However, if a foreign body cannot be accommodated within the hood, the risk of gastrointestinal injury exists. Herein, we report a safe retrieval technique using a novel endoscopic distal attachment specifically designed for foreign body retrieval (EndoFlower; Fujifilm Medical Corporation, Tokyo, Japan).

**Fig. 1:**
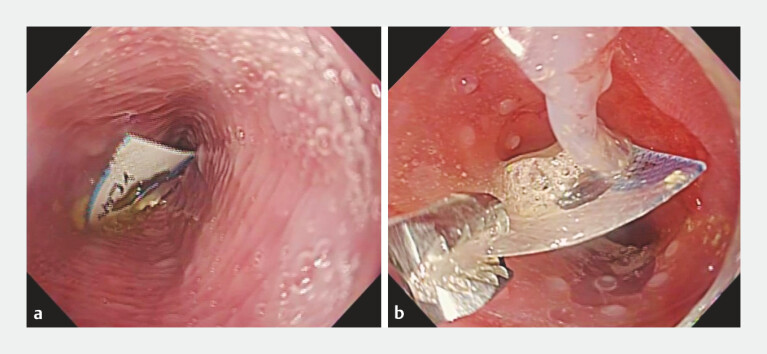
A case of accidental ingestion of a press-through package (PTP) sheet (Case 1). **a** An endoscopic image showing an impacted PTP sheet in the mid-esophagus. **b** Retrieval of the PTP sheet covered with the novel endoscopic distal attachment.

Case 1 ( Fig. 1 ): A 75-year-old woman accidentally ingested a press-through package (PTP) sheet.

**Fig. 2:**
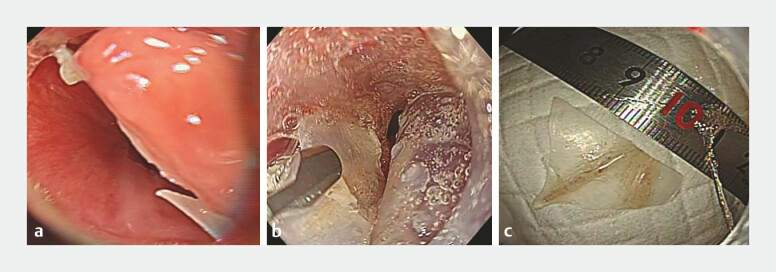
A case of accidental ingestion of a fish-bone (Case 2). **a** An endoscopic image showing a fish-bone impacted at the entrance of the esophagus. **b** Retrieval of the fish-bone covered with the novel endoscopic distal attachment. **c** Fish bones after collection.

Case 2 ( Fig. 2 ): A 57-year-old woman accidentally ingested a fish-bone.

**Fig. 3:**
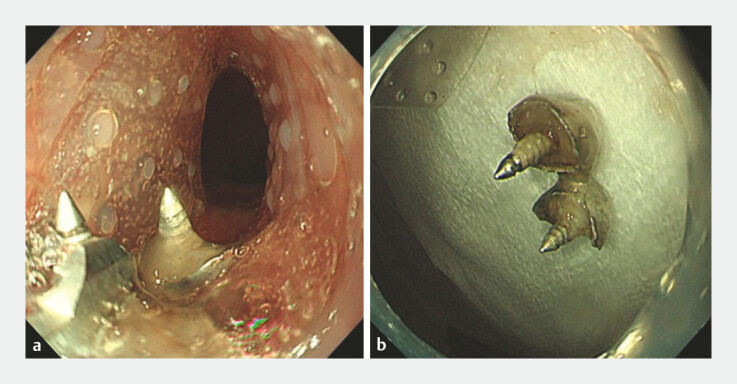
A case of accidental ingestion of a denture (Case 3). **a** Retrieval of the denture covered with the novel endoscopic distal attachment. **b** Dentures after collection.

Case 3 ( Fig. 3 ): A 92-year-old woman had accidentally ingested a denture.

In all cases, the foreign body was successfully retrieved using an endoscope equipped with a novel attachment, without causing any gastrointestinal injury ( Video 1 ).

Safe retrieval of gastrointestinal foreign bodies using a novel endoscopic distal attachment.Video 1

**Fig. 4:**
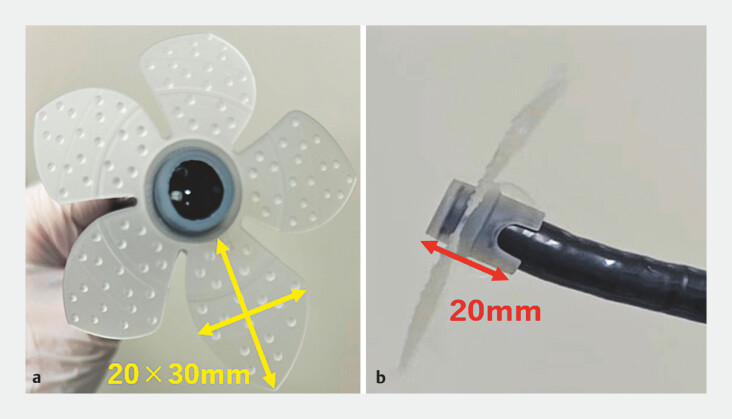
Novel endoscopic distal attachment specifically designed for foreign body retrieval. **a** and **b** The attachment comprising five silicone, flower-shaped flaps. This attachment has a total length of 20 mm and consists of five 20 × 30 mm flaps.

**Fig. 5:**
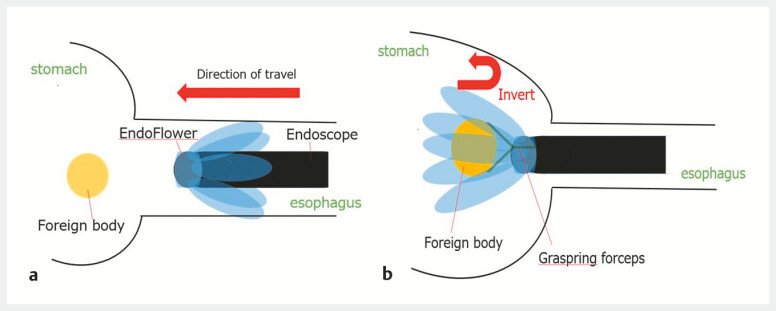
A schematic diagram of EndoFlower. **a** Inserting of the EndoFlower. **b** Removal of foreign objects using EndoFlowers.

The novel attachment is a dedicated foreign-body retrieval device composed of five silicone flaps in a flower-shaped design. This attachment has a total length of 20 mm and consists of five 20 × 30mm flaps ( Fig. 4 ). During endoscope insertion, the flaps are folded opposite to the direction of scope advancement to avoid obstructing the visual field. Although esophageal inversion of the flaps is sometimes possible, it was difficult in the present three cases; therefore, they were inverted in the stomach to envelop the foreign body ( Fig. 5 ).

This attachment may be a safe and effective option for foreign body retrievals.

Endoscopy_UCTN_Code_CCL_1AB_2AF
